# Similarities between the Epstein-Barr Virus (EBV) Nuclear Protein EBNA1 and the Pioneer Transcription Factor FoxA: Is EBNA1 a “Bookmarking” Oncoprotein that Alters the Host Cell Epigenotype?

**DOI:** 10.3390/pathogens1010037

**Published:** 2012-09-17

**Authors:** Hans Helmut Niller, Janos Minarovits

**Affiliations:** 1 Institute for Medical Microbiology and Hygiene, University of Regensburg, Franz-Josef-Strauß Allee 11, Regensburg D-93053, Germany; 2 Microbiological Research Group, National Center for Epidemiology, Piheno u. 1., Budapest H-1529, Hungary; E-Mail: minimicrobi@hotmail.com

**Keywords:** DNA-looping, CpG-methylation, histone, chromatin, *oriP*

## Abstract

EBNA1, a nuclear protein expressed in all EBV-associated neoplasms is indispensable for the maintenance of the viral episomes in latently infected cells. EBNA1 may induce genetic alterations by upregulating cellular recombinases, production of reactive oxygen species (ROS) and affecting p53 levels and function. All these changes may contribute to tumorigenesis. In this overview we focus, however, on the epigenetic alterations elicited by EBNA1 by drawing a parallel between EBNA1 and the FoxA family of pioneer transcription factors. Both EBNA1 and FoxA induce local DNA demethylation, nucleosome destabilization and bind to mitotic chromosomes. Local DNA demethylation and nucleosome rearrangement mark active promoters and enhancers. In addition, EBNA1 and FoxA, when associated with mitotic chromatin may “bookmark” active genes and ensure their reactivation in postmitotic cells (epigenetic memory). We speculate that DNA looping induced by EBNA1-EBNA1 interactions may reorganize the cellular genome. Such chromatin loops, sustained in mitotic chromatin similarly to the long-distance interactions mediated by the insulator protein CTCF, may also mediate the epigenetic inheritance of gene expression patterns. We suggest that EBNA1 has the potential to induce patho-epigenetic alterations contributing to tumorigenesis.

## 1. Introduction

Epstein-Barr virus (EBV), a herpesvirus widespread in human populations, replicates in oropharyngeal epithelial cells and establishes lifelong latent infection in resting memory B cells. Although the primary infection is usually inapparent, the association of EBV with a series of lymphomas and carcinomas and its potential role in the pathogenesis of certain autoimmune diseases resulted in a detailed description of the viral latency products and their host cell dependent expression patterns (EBV latency types) (reviewed in [1,2]). EBV-encoded nuclear proteins (EBNAs), transmembrane proteins (latent membrane proteins, LMPs) and non-translated RNAs (EBERs and microRNAs) were implicated both in tumorigenesis and *in vitro* immortalization of human B lymphocytes. The nuclear antigen EBNA1 is indispensable for the maintenance of viral latency due to its involvement in the duplication, partitioning and maintenance of the viral episomes [3]. Most research focused on how EBNA1 attracts the cellular replication machinery to *oriP*, the latent origin of replication, and how EBNA1 mediates nonrandom segregation of the viral episomes. Thus, EBNA1 was not considered to be a typical viral oncoprotein that alters the behaviour of its target cells: such a role was usually attributed to other EBV latency products (LMP1, EBNA2, EBERs, microRNAs). The contribution of EBNA1 to neoplastic development was thought to be the maintenance of the viral episomes within the cells, permitting thereby the expression of latent proteins and RNAs directly involved in oncogenesis. Unexpectedly, however, certain observations suggested a more direct role for EBNA1 in the initiation and progression of malignant neoplasms and pointed to the potential importance of EBNA1-induced genetic alterations in tumorigenesis. Srinivas and Sixbey demonstrated that EBNA1 could induce the expression of the V(D)J recombinase-activating genes RAG1 and RAG2 in an EBV negative Burkitt’s lymphoma (BL) cell line [4]. Because RAG1 and RAG2 were implicated in eliciting chromosome translocations in B-cell neoplasms, these data suggested that EBNA1 may promote genomic instability resulting in cytogenetic alterations and lymphomagenesis. In addition, EBNA1 up-regulated NOX2, the catalytic subunit of the NADPH oxidase involved in the production of reactive oxygen species (ROS), possibly through an EBNA1-binding site the within the NOX2-promoter, and induced chromosomal alterations and DNA double-strand breaks *in vitro* [5,6]. Sivachandran *et al*. speculated that a decreased apoptotic response and impaired DNA repair associated with the downregulation of p53 levels by EBNA1 [7] as well as impaired acetylation of p53 due to the disruption of promyelocytic leukemia nuclear bodies (PML NBs) by EBNA1 also contribute to the development of EBV-associated neoplasms [8,9].

Recently, the genetic theory of carcinogenesis that dominated the field of cancer research for a long time was supplemented with epigenetic models, and it was observed that viral oncoproteins regularly cause epigenetic dysregulation in neoplastic cells (reviewed in [10,11]). In this review we focus on the epigenetic changes elicited by EBNA1 and compare them to the exciting new observations as to the epigenetic marks left on the chromatin by pioneer transcription factors.

## 2. Pioneer Transcription factors: The FoxA Family

In mammals, pioneer transcription factors control organogenesis of liver, lung, pancreas, prostate and midbrain [12,13]. These DNA binding proteins may enable cellular reprogramming by binding to enhancer regions even in highly compacted chromatin regions [14] (reviewed in [15]).

FoxA proteins, the prototype pioneer factors that belong to the forkhead box (Fox) family are thought to open chromatin through replacement of linker histone H1, a structurally similar winged helix domain protein [14,16] (reviewed in [17,18]). Due to their high nucleosome-binding affinity, FoxA1 and FoxA2 move more slowly in nuclei than other classes of transcription factors (c-Myc, GATA-4, NF-1 and HMGB1) [19]. FoxA binding increases the accessibility of other transcription factors to the neighboring regulatory sequences that flank FoxA recognition sites. In addition, binding of FoxA is associated with the maintenance or creation of an epigenetic mark, local CpG hypomethylation as well [12]. This means that FoxA binding either prevents *de novo* cytosine methylation at CpG dinucleotides recognized by DNA methyltransferases or induces active or passive demethylation of methylcytosines within or in the vicinity of its recognition sequence. Sérandour *et al*. argued that in a neural differentiation model the establishment of transcriptional competence at FOXA1-dependent enhancers is associated with an epigenetic switch that involves both DNA demethylation and induction of histone H3 mono- and dimethylation at lysine 4 (H3K4me1, H3K4me2) [20]. Because retinoic acid induced differentiation of P19 embryonal carcinoma cells does not favour cell division, one may speculate that FoxA1 binding probably induced active, enzymatic demethylation of certain methylcytosine residues in the vicinity of its recognition sequences. Passive demethylation, based on the inhibition of the maintenance DNA methyltransferase DNMT1 during the S phase of the cell cycle depends on cell proliferation and seems to be a less plausible mechanism in differentiating cells. The capacity to demethylate *in vitro* pre-methylated plasmids carrying cloned enhancer sequences depends on the cell type. Such enhancers either remain silent and methylated in differentiated cells unless the repressive Mi-2/NurD chromatin remodeling complex is depleted, or, as in certain embryonic stem cells and induced pluripotent stem cells, they may undergo demethylation despite the absence of active transcription [21,22].

In embryonic stem cells silent tissue-specific enhancers were marked by windows of unmethylated CpG dinucleotides, some of them located within pioneer factor binding sites [21]. These findings were in harmony with the idea that in embryonic stem cells a permissive chromatin structure is assembled at the enhancers of inactive tissue-specific promoters. Such preexisting protein-DNA interactions or „enhancer marks” may prevent the formation of a repressive chromatin structure during differentiation and permit the activation of silent promoters at a subsequent developmental stage in response to tissue-inductive signals, steroid hormones, other stimuli or pathological alterations (metaplasia, tumorigenesis) [12,21,23,24]. It is worthy to note that FoxA1, in concert with the transcription factors MYBL2 and CREB1, also induced histone H3 acetylation, a histone modification characteristic for active chromatin, and facilitates nucleosome disruption in castration-resistant prostate cancer [25]. 

The long terminal repeat sequence of mouse mammary tumor virus (MMTV LTR) also carries binding sites for FoxA, and Holmqvist *et al*. observed that attachment of FoxA1 resulted in nucleosome rearrangement and affected the activity of the MMTV promoter [26]. Furthermore, forkhead box proteins FoxO1 and FoxA (also called hepatocyte nuclear factor 3, HNF3), are involved in the regulation of hepatitis B virus transcription in infected liver cells [27] (reviewed in [28]).

## 3. “Bookmarking” the Mitotic Chromatin: A Novel form of Epigenetic Memory

Both FoxA1 as well as FoxI1, another member of the Fox family expressed in zebrafish, remain bound to chromatin even in mitotic chromosomes [12,29], suggesting that their binding to DNA might serve as an independent epigenetic mark. Thus, similarly to the complexes of Polycomb and Trithorax family proteins and DNA methylation [30], chromatin-bound pioneer factors may also ensure the inheritance of gene expression patterns. Indeed, Zaret *et al*. speculated that retaining of a pioneer factor (so called „bookmarking” protein) in mitotic chromatin may help to “remember” postmitotic daughter cells the gene expression pattern of the parental cell by facilitating a more rapid or synchronous activation of the FoxA target genes following chromosome decondensation [12]. The concept of bookmarking genes for activation in condensed mitotic chromosomes was introduced by John and Workman [31]. It turned to be that bookmarking is mediated by protein-DNA interactions that alter chromatin structure. Xing *et al*. demonstrated that binding of the transcription factor HSF2 (heat shock factor 2) to the promoter region of the *hsp70i *gene in mitotic cells prevents compaction by the recruitment of protein phosphatase 2A (PP2A) that inactivates condensin complexes [32] (reviewed in [33]). During mitosis, HSF2 bookmarks the promoters of other heat shock genes (*hsp90*, *hsp27*) and the *c-fos* proto-oncogene promoter, too [34]. Similarly to HSF2, TBP (TATA-binding protein) was found to be retained at many chromosomal sites during mitosis. TBP recruited PP2A and interacted with condensin, suggesting that it may contribute to gene bookmarking [35] (reviewed in [36]). Hepatocyte nuclear factor-1β (HNF-1β), a transcription factor deleted in polycystic kidney disease, remains associated with mitotic chromosomes as well [37]. HNF-1β was suggested to act as a bookmarking factor reopening the chromatin of its target genes after mitotic silencing [37]. Genes highly transcribed during interphase retained the Trithorax protein MLL (mixed lineage leukemia) at their promoters during mitosis [38]. This observation implies that mitotic bookmarking contributes to gene regulation by Trithorax complexes by accelerating transcriptional reactivation following mitosis. Because MLL is a histone methyltransferase which catalyzes H3K4 tri-methylation, these data are consistent with the finding that H3K4me3, a mark of euchromatin in the G1 phase of the cell cycle, remained associated with the condensed chromatin of mitotic chromosomes as well [39]. 

## 4. “Bookmarking” of Active Promoters by a Variant Histone and Altered Nucleosome Occupancy

Recently, the histone variant H2A.Z, localized in a hyperacetylated form near the transcriptional start site (TSS) of active genes in interphase cells, was found to be associated, although in a deacetylated form, with mitotic chromosomes in chicken cells [40] and M phase chromatin of human cells [41]. Kelly *et al*. suggested that H2A.Z may mark certain gene sets for rapid re-expression following mitotic exit and destabilize nucleosomes located in the vicinity of transcriptional start sites, allowing nucleosome sliding either toward the TSS, that blocks transcription factor and RNA polymerase II binding during mitosis, or away from the TSS that may permit promoter activation after chromosome decondensation [41]. Kelly and Jones speculated that nucleosomal rearrangement combined with the H2A.Z mark and modifications of histone H3 (H3K4me3, and phosphorylation at serine 10, H3S10P) allows the nucleosome to maintain an active configuration in the vicinity of silent promoters in mitotic chromatin and permits a rapid reactivation of the marked promoters following mitotic exit [42]. Thus, marking of active promoters by altered nucleosome occupancy at the TSS may serve as a novel, general epigenetic mechanism ensuring gene reactivation and restoration of cell type specific gene expression patterns in the daughter cells following mitosis. 

## 5. Factors other than FoxA: Association with Mitotic Chromosomes

Factors repressing transcription may also associate with mitotic chromosomes. Runx2, a runt-related transcription factor, acts as a repressor of RNA polymerase I mediated ribosomal RNA (rRNA) synthesis, and it is retained associated with rRNA genes at nucleolar organizing regions within the condensed mitotic chromosomes [43]. It is noteworthy that Runx2, a regulator of osteogenesis, also associated with the RNA polymerase II transcribed promoters of its target genes in mitotic chromosomes and established a transcriptionally poised chromatin structure at the target gene promoters [44]. This silent chromatin thought to be ready for transcription contained a basal level of acetylated histone H4 and a constitutively high level of histone H3 dimethylated at lysine 4. One may wonder, however, whether–in mitotic chromosomes–Runx2 also remains associated with the promoters of RNA polymerase II transcribed genes that happen to be switched off by Runx2 binding [45]. This possibility remains to be investigated. In a recent live cell imaging study, Pockwinse *et al.* demonstrated that the binding affinity of Runx2 apparently increased as chromosomes condensed, possibly due to phosphorylation during mitosis [46]. Such a mechanism may affect Runx2 proteins both in transcriptionally active or repressive chromatin. 

PU.1, a transcription factor interacting with lineage-specific enhancers in B cells and myeloid cells appears to maintain a high level of histone H3 lysine 4 mono-methylation (H3K4me1) at its targets and cooperates with stimulus-responsive transcription factors in enhancer activation [47,48]. PU.1 belongs to the ets family of regulatory proteins and the structure of its conserved ETS DNA binding domain is similar to the build up of “winged” helix-turn-helix proteins [49]. Whether PU.1 can replace histone H1 at enhancers, similarly to FoxA, remains to be clarified. It is worthy to note, however, that in addition to establishing a permissive chromatin structure at myeloid or B cell specific genes, PU.1 may also create a repressive chromatin structure to extinguish the activity of genes involved in erythroid differentiation. Downregulation of the alternative gene expression program is achieved by binding of PU.1 to GATA-1, a pioneer factor indispensable for erythroid differentiation. PU.1 blocks GATA-1 DNA binding by interacting with the C-terminal zinc finger of GATA-1 [50]. In addition, PU.1 organizes a repressor protein complex at GATA-1 regulated promoters [51]. The repressive chromatin contains heterochromatin protein 1 (HP1) and modified histone H3 methylated at lysine-9. 

PBX1 (pre-B-cell leukemia homeobox 1), a homeodomain protein, interacts with and affects the DNA-binding specificity and affinity of Hox (homeotic) proteins which are governing the choice between alternative developmental pathways [52,53]. Recently PBX1, involved in diverse developmental processes [54], was characterized as a novel pioneer factor that may open chromatin at specific genomic locations and recruit estrogen receptor alpha (ERα) to estrogen responsive genes in breast cancer cells [55]. Magnani *et al*. argued that a specific epigenetic signature, histone H3 di-methylated at lysine 4 (H3K4me2), is preferentially recognized by PBX1. It is worthy to note that also the pioneer factor FoxA1 recruits ERα to H3K4me2-enriched regulatory elements in breast cancer cells [56], but only PBX1 expression correlated with metastasis formation and development of resistance to endocrine therapies in patients with ERα positive breast cancer [55]. 

## 6. Chromatin Loops Maintained in Mitotic Chromatin

In addition to pioneer factors, CTCF (CCCTC-binding factor), an insulator protein separating chromatin domains and mediating higher order chromatin interactions (looping) is also bound to mitotic chromatin [57]. Burke *et al*. suggested that CTCF binding may represent a novel form of epigenetic memory, because it maintains distinct long-distance interactions in mitotic chromosomes that may facilitate the reestablishment of other higher order interactions lost in mitotic chromatin, after cell division [57]. 

## 7. EBNA1: Anchoring of EBV Episomes and Looping DNA

Epstein-Barr virus (EBV), a human gammaherpesvirus, is associated with a series of malignant tumors (reviewed in [1]). EBV replicates in the epithelial and lymphoid cells of the oropharynx and establishes latent infection in memory B cells. The lymphomas and carcinomas associated with EBV also carry latent viral episomes expressing only a subset of the viral genes. The only EBV-encoded nuclear antigen invariably present in EBV-associated neoplasms is EBNA1 that binds to *oriP*, the latent origin of EBV replication and tethers the viral episomes to the nuclear matrix in interphase cells and to metaphase chromosomes during mitosis (reviewed in [58]). 

Episomal maintenance in cells carrying latent EBV genomes is achieved by EBNA1 that binds directly to multiple sequence specific binding sites within *oriP *and binds directly or indirectly to AT-rich DNA sequences of host cell chromosomes [59,60,61]. Whereas EBNA1 homodimers bind to *oriP via* the carboxy-terminal DNA-binding domain that contacts both the major and the minor groove of the DNA [62,63], association with cellular chromosomes is mediated by three amino-terminal EBNA1 domains [64], two of them containing repeats of glycine and arginine (GR repeats), termed AT hooks [60]. The second GR-repeat is mostly responsible for chromosomal attachment [61,65,66]. GR repeat 2 also interacts with the cellular protein EBP2 (EBNA1 binding protein 2) that ensures a tight chromosomal association and mitotic segregation of EBV genomes [61,67,68]. Based on experiments with *oriP* containing plasmids, Sears *et al*. argued that metaphase chromosome tethering of the episomes by EBNA1 may create an opportunity for the cellular origin recognition complex (ORC), located at AT-rich DNA regions of cellular DNA, to interact with and be loaded onto *oriP* [60]. Replacement of the basic chromosome-associating domains of EBNA1 by either amino acids 1-90 of the high-mobility-group protein HMG-I, full-length HMG1a, or substituting them with the basic protein histone H1 resulted in hybrid proteins enabling the EBNA1 C-terminus to bind to mitotic chromosomes [59,69]. 

EBNA1 not only governs the replication and segregation of the viral episomes, but by binding to a repetitive sequence (family of repeats, FR) within *oriP* it also transactivates the latent EBV promoters Cp and LMP1p [70,71], presumably *via* one of its looping/linking domains (residues 325–376; [72,73,74]. The EBNA1 looping/linking/transactivating domain contributes to the maintenance of the episomal EBV genomes in latently infected cells (reviewed in [3]). Association between the looping/linking domains of EBNA1 dimers or monomers bound to distant regions may result in looping out of the intervening DNA [73,75,76,77]. We suggest that certain higher order chromatin structures (loops) created by EBNA1-EBNA1 interactions may be sustained in mitotic chromosomes and form the basis of a novel type of epigenetic memory that involves a viral protein. Thus, EBNA1 bound to its recognition sequences in the host cell genome may alter the architecture and interactions of certain genomic regions and ensure the epigenetic inheritance of such a novel higher order structure from cell generation to cell generation.

## 8. EBNA1: Marking Cellular Genes

In addition to interacting with EBV genomes, EBNA1 also binds diverse sequence motifs close to the transcriptional start sites of a series of cellular genes in Raji Burkitt lymphoma (BL) cells [78]. EBNA1 binding sites were found in LINE1 retrotransposons as well. Lu *et al*. observed that the consensus sequence for cellular EBNA1 binding sites differs from that of the binding sites within the EBV genome, located at FR and the dyad symmetry (DS) element within *oriP*, and at the Q promoter (Qp). This suggests that the cellular sites may bind EBNA1 indirectly [78]. Transfection of the EBNA1 gene into the EBV negative BL line BJAB and the epithelial cell line 293 also revealed a DNA motif at EBNA1 regulated cellular promoters that differed from the viral binding sites [79]. Several EBNA1-binding sites were found in the cellular genome of 721 lymphoblastoid cells and in the viral genomes of KSHV and Herpesvirus papio. A rep*-like sequence from the Herpesvirus papio genome which binds EBNA1 functioned as an origin of DNA replication in Raji cells [80]. The consensus motif for EBNA1 binding defined by Dresang *et al*. was similar to Motif 2 found by Lu *et al*. [78,80]. The partial overlap and differences between the sequence motifs defined by these three studies [78,79,80] may be due to the use of different cell lines. An additional computational approach identified a set of 40 FR-like sequences within the human genome which may specifically bind EBNA1. The functional significance of these potential EBNA1 binding sites needs to be further explored [81]. An EBNA1 deletion mutant leaving both GR repeats intact, but deleting the small unique region 1 (UR1) just next to GR repeat 1, defined a novel transcriptional transactivation domain within EBNA1 [58]. ΔUR1 mutant viruses segregated the plasmid maintenance and transcriptional activation functions of EBNA1. While supporting DNA binding and plasmid maintenance, the UR1 deletion mutant was not able to transcriptionally activate the C-promoter anymore. Thus, the Wp to Cp switch did not occur in cells infected with the respective ΔUR1 viruses, and the infected cells were not transformed, but ceased growing shortly after infection [82]. The UR1 peptide, which is conserved among EBNA1 orthologs of other γ-herpesviruses and is also found in the catalytic domains of some DNA polymerase δ enzymes, binds and coordinates Zn^2+^ ions and contributes to EBNA1 dimerization. Furthermore, the UR1 transactivation function of EBNA1 on Cp is sensitive to oxidation levels and works best under hypoxic conditions [83]. Whether the UR1 peptide plays a role in activating cellular genes needs further studies.

## 9. EBNA1: Displacement of Nucleosomes and Induction of Site-Specific Demethylation

In cells carrying latent EBV episomes, viral DNA synthesis is initiated once per cell cycle typically at the DS element containing four EBNA1 binding sites (reviewed in [3]). EBNA1 is capable of interacting with the DS element, even if it is assembled into a nucleosome core particle, and EBNA1 binding results in nuclesosome destabilization and displacement [84]. Access to the EBNA1 binding sites at DS and FR by EBNA1 does not require ATP-dependent chromatin-remodeling factors [85]. In contrast, the nucleosomes flanking DS undergo chromatin remodeling mediated by the SNF2h (sucrose nonfermenting 2 homolog) ATPase, in parallel with histone deacetylation. This event happens at the G1/S-border of the cell cycle after the cellular minichromosome maintenance (MCM) helicase complex is loaded to the four EBNA1 homodimers of the DS element associated with the origin recognition complex (ORC), another component of the cellular replicative machinery [86]. Binding of parts of the MCM complex after mitosis licences *oriP* firing in the ensuing S phase [87] (reviewed in [3]). Binding of EBNA1 to its recognition sites was stimulated by the recruitment of the cellular deubiquitylating enzyme USP7 which cleaves monoubiquitin from histone H2B. Thus, by recruiting USP7, EBNA changes the histone ubiquitylation state of the chromatin at oriP [88].

EBNA1 is capable of binding to both unmethylated and methylated *oriP* sequences. Attachment of EBNA1 to its binding sites carrying methylated CpG dinucleotides results in a simultaneous site-specific demethylation both in FR and DS, provided that the *oriP* sequences are replicating [89]. The site-specific demethylation affects only methylcytosines located within EBNA1 binding sites: adjacent methylcytosines in the region, situated near to but outside EBNA1 recognition sequences remain methylated. Thus, EBNA1 binding can specify demethylation sites. Hsieh suggested that EBNA1 binding interferes with the function of the maintenance DNA methyltransferase (DNMT1) that copies the DNA methylation pattern of the parental strand to the daughter strand after DNA replication, resulting in passive demethylation of the first DNA strand at *oriP*, whereas active demethylation, mediated by a demethylase enzyme recruited to hemimethylated DNA may occur on the second strand of *oriP* [89]. High affinity protein binding to a methylated DNA sequence located outside of an origin of DNA replication may also result in site-specific demethylation [90]. 

## 10. EBNA1: Reprogramming the Epigenome?

Binding of EBNA1 to metaphase chromosomes [91,92,93] and cellular promoters [78,79] raises the possibility that EBNA1 acts like a bookmarking protein that affects the expression of its cellular target genes in a heritable manner. We suggest that epigenetic inheritance of the EBNA1 dysregulated cellular gene expression pattern may be ensured by local demethylation and nucleosomal rearrangements induced by EBNA1 at its cellular binding sites (Table 1). Although EBNA1 is regularly expressed in EBV-associated neoplasms and *in vitro* transformed lymphoblastoid cell lines (LCLs), its role in oncogenesis was perceived mainly as an indirect one, *i.e.*, maintenance of EBV episomes in the tumor cells, thereby permitting the expression of other viral proteins and non-translated viral RNAs that initiate and maintain malignant transformation. Expression of EBNA1 induced B cell neoplasia in certain transgenic mouse lines [94] but not in others [95,96]. In spite of these contradictory data, a role for EBNA1 as an oncoprotein in the generation of human neoplasms cannot be excluded at present. The functional similarities between the pioneer factor FoxA and EBNA1 (Table 1) as well as CTCF and EBNA1 suggest that EBNA1 is potentially capable for epigenetic reprogramming of EBV target cells which may result in tumorigenesis. 

**Table 1:** Interactions of the pioneer transcription factor FoxA and the viral oncoprotein EBNA1 with epigenetic regulatory mechanisms.

Epigenetic mechanism	Phenomenon elicited by	
	FoxA	EBNA1
DNA methylation	Demethylation	Demethylation
Histone acetylation	Upregulationhistone H3	?
Histone methylation	?	?
Polycomb/Trithorax complexes	?	?
Binding to mitotic chromosomes	“Bookmarking”?	“Bookmarking”?
Nucleosome rearrangement	Alteration of a hormone-dependent sub-nucleosome complex at the MMTV LTR	Nucleosome destabilization at *oriP *of EBV episomes
DNA looping	?	EBNA1-EBNA1 binding may link distinct DNA regions in viral and cellular genomes (epigenetic memory?)

## References

[1] Niller H.H., Wolf H., Minarovits J., Minarovits J., Gonczol E., Valyi-Nagy T. (2007). Epstein-Barr Virus. Latency Strategies of Herpesviruses.

[2] Niller H.H., Wolf H., Minarovits J. (2008). Regulation and dysregulation of Epstein-Barr virus latency: Implications for the development of autoimmune diseases. Autoimmunity.

[3] Lindner S.E., Sugden B. (2007). The plasmid replicon of Epstein-Barr virus: Mechanistic insights into efficient, licensed, extrachromosomal replication in human cells. Plasmid.

[4] Srinivas S.K., Sixbey J.W. (1995). Epstein-Barr virus induction of recombinase-activating genes RAG1 and RAG2. J. Virol..

[5] Gruhne B., Sompallae R., Marescotti D., Kamranvar S.A., Gastaldello S., Masucci M.G. (2009). The Epstein-Barr virus nuclear antigen-1 promotes genomic instability via induction of reactive oxygen species. Proc. Natl. Acad. Sci. USA.

[6] Cao J.Y., Mansouri S., Frappier L. (2012). Changes in the nasopharyngeal carcinoma nuclear proteome induced by the EBNA1 protein of Epstein-Barr virus reveal potential roles for EBNA1 in metastasis and oxidative stress responses. J. Virol..

[7] Saridakis V., Sheng Y., Sarkari F., Holowaty M.N., Shire K., Nguyen T., Zhang R.G., Liao J., Lee W., Edwards A.M. (2005). Structure of the p53 binding domain of HAUSP/USP7 bound to Epstein-Barr nuclear antigen 1 implications for EBV-mediated immortalization. Mol. Cell..

[8] Sivachandran N., Sarkari F., Frappier L. (2008). Epstein-Barr nuclear antigen 1 contributes to nasopharyngeal carcinoma through disruption of PML nuclear bodies. PLoS Pathog..

[9] Sivachandran N., Dawson C.W., Young L.S., Liu F.F., Middeldorp J., Frappier L. (2012). Contributions of the Epstein-Barr virus EBNA1 protein to gastric carcinoma. J. Virol..

[10] Minarovits J. (2009). Microbe-induced epigenetic alterations in host cells: The coming era of patho-epigenetics of microbial infections. A review. Acta Microbiol. Immunol. Hung..

[11] Niller H.H., Banati F., Ay E., Minarovits J., Minarovits J., Niller H.H. (2012). Epigenetic Changes in Virus-Associated Neoplasms. Patho-Epigenetics of Disease.

[12] Zaret K.S., Watts J., Xu J., Wandzioch E., Smale S.T., Sekiya T. (2008). Pioneer factors, genetic competence, and inductive signaling: Programming liver and pancreas progenitors from the endoderm. Cold Spring Harb. Symp. Quant. Biol..

[13] Kaestner K.H. (2010). The FoxA factors in organogenesis and differentiation. Curr. Opin. Genet. Dev..

[14] Cirillo L.A., Lin F.R., Cuesta I., Friedman D., Jarnik M., Zaret K.S. (2002). Opening of compacted chromatin by early developmental transcription factors HNF3 (FoxA) and GATA-4. Mol. Cell.

[15] Zaret K.S., Carroll J.S. (2011). Pioneer transcription factors: Establishing competence for gene expression. Genes Dev..

[16] Clark K.L., Halay E.D., Lai E., Burley S.K. (1993). Co-crystal structure of the HNF-3/fork head DNA-recognition motif resembles histone H5. Nature.

[17] Zaret K.S., Caravaca J.M., Tulin A., Sekiya T. (2010). Nuclear mobility and mitotic chromosome binding: Similarities between pioneer transcription factor FoxA and linker histone H1. Cold Spring Harb. Symp. Quant. Biol..

[18] Hirai H., Tani T., Kikyo N. (2010). Structure and functions of powerful transactivators: VP16, MyoD and FoxA. Int. J. Dev. Biol..

[19] Sekiya T., Muthurajan U.M., Luger K., Tulin A.V., Zaret K.S. (2009). Nucleosome-binding affinity as a primary determinant of the nuclear mobility of the pioneer transcription factor FoxA. Genes Dev..

[20] Serandour A.A., Avner S., Percevault F., Demay F., Bizot M., Lucchetti-Miganeh C., Barloy-Hubler F., Brown M., Lupien M., Metivier R. (2011). Epigenetic switch involved in activation of pioneer factor FOXA1-dependent enhancers. Genome Res..

[21] Xu J., Pope S.D., Jazirehi A.R., Attema J.L., Papathanasiou P., Watts J.A., Zaret K.S., Weissman I.L., Smale S.T. (2007). Pioneer factor interactions and unmethylated CpG dinucleotides mark silent tissue-specific enhancers in embryonic stem cells. Proc. Natl. Acad. Sci. USA.

[22] Xu J., Watts J.A., Pope S.D., Gadue P., Kamps M., Plath K., Zaret K.S., Smale S.T. (2009). Transcriptional competence and the active marking of tissue-specific enhancers by defined transcription factors in embryonic and induced pluripotent stem cells. Genes Dev..

[23] Smale S.T. (2010). Pioneer factors in embryonic stem cells and differentiation. Curr. Opin. Genet. Dev..

[24] Watts J.A., Zhang C., Klein-Szanto A.J., Kormish J.D., Fu J., Zhang M.Q., Zaret K.S. (2011). Study of FoxA pioneer factor at silent genes reveals Rfx-repressed enhancer at Cdx2 and a potential indicator of esophageal adenocarcinoma development. PLoS Genet..

[25] Zhang C., Wang L., Wu D., Chen H., Chen Z., Thomas-Ahner J.M., Zynger D.L., Eeckhoute J., Yu J., Luo J. (2011). Definition of a FoxA1 Cistrome that is crucial for G1 to S-phase cell-cycle transit in castration-resistant prostate cancer. Cancer Res..

[26] Holmqvist P.H., Belikov S., Zaret K.S., Wrange O. (2005). FoxA1 binding to the MMTV LTR modulates chromatin structure and transcription. Exp. Cell Res..

[27] Shlomai A., Shaul Y. (2009). The metabolic activator FOXO1 binds hepatitis B virus DNA and activates its transcription. Biochem. Biophys. Res. Commun..

[28] Bar-Yishay I., Shaul Y., Shlomai A. (2011). Hepatocyte metabolic signalling pathways and regulation of hepatitis B virus expression. Liver Int..

[29] Yan J., Xu L., Crawford G., Wang Z., Burgess S.M. (2006). The forkhead transcription factor FoxI1 remains bound to condensed mitotic chromosomes and stably remodels chromatin structure. Mol. Cell Biol..

[30] Blomen V.A., Boonstra J. (2011). Stable transmission of reversible modifications: Maintenance of epigenetic information through the cell cycle. Cell Mol. Life Sci..

[31] John S., Workman J.L. (1998). Bookmarking genes for activation in condensed mitotic chromosomes. Bioessays.

[32] Xing H., Wilkerson D.C., Mayhew C.N., Lubert E.J., Skaggs H.S., Goodson M.L., Hong Y., Park-Sarge O.K., Sarge K.D. (2005). Mechanism of hsp70i gene bookmarking. Science.

[33] Sarge K.D., Park-Sarge O.K. (2005). Gene bookmarking: Keeping the pages open. Trends Biochem. Sci..

[34] Wilkerson D.C., Skaggs H.S., Sarge K.D. (2007). HSF2 binds to the Hsp90, Hsp27, and c-Fos promoters constitutively and modulates their expression. Cell Stress Chaperones.

[35] Xing H., Vanderford N.L., Sarge K.D. (2008). The TBP-PP2A mitotic complex bookmarks genes by preventing condensin action. Nat. Cell Biol..

[36] Sarge K.D., Park-Sarge O.K. (2009). Mitotic bookmarking of formerly active genes: Keeping epigenetic memories from fading. Cell Cycle.

[37] Verdeguer F., Le Corre S., Fischer E., Callens C., Garbay S., Doyen A., Igarashi P., Terzi F., Pontoglio M. (2010). A mitotic transcriptional switch in polycystic kidney disease. Nat. Med..

[38] Blobel G.A., Kadauke S., Wang E., Lau A.W., Zuber J., Chou M.M., Vakoc C.R. (2009). A reconfigured pattern of MLL occupancy within mitotic chromatin promotes rapid transcriptional reactivation following mitotic exit. Mol. Cell.

[39] Mishra B.P., Ansari K.I., Mandal S.S. (2009). Dynamic association of MLL1, H3K4 trimethylation with chromatin and Hox gene expression during the cell cycle. FEBS J..

[40] Bruce K., Myers F.A., Mantouvalou E., Lefevre P., Greaves I., Bonifer C., Tremethick D.J., Thorne A.W., Crane-Robinson C. (2005). The replacement histone H2A.Z in a hyperacetylated form is a feature of active genes in the chicken. Nucleic Acids Res..

[41] Kelly T.K., Miranda T.B., Liang G., Berman B.P., Lin J.C., Tanay A., Jones P.A. (2010). H2A.Z maintenance during mitosis reveals nucleosome shifting on mitotically silenced genes. Mol. Cell.

[42] Kelly T.K., Jones P.A. (2011). Role of nucleosomes in mitotic bookmarking. Cell Cycle.

[43] Young D.W., Hassan M.Q., Pratap J., Galindo M., Zaidi S.K., Lee S.H., Yang X., Xie R., Javed A., Underwood J.M. (2007). Mitotic occupancy and lineage-specific transcriptional control of rRNA genes by Runx2. Nature.

[44] Young D.W., Hassan M.Q., Yang X.Q., Galindo M., Javed A., Zaidi S.K., Furcinitti P., Lapointe D., Montecino M., Lian J.B. (2007). Mitotic retention of gene expression patterns by the cell fate-determining transcription factor Runx2. Proc. Natl. Acad. Sci. USA.

[45] Tandon M., Gokul K., Ali S.A., Chen Z., Lian J., Stein G.S., Pratap J. (2012). Runx2 mediates epigenetic silencing of the bone morphogenetic protein-3B (BMP-3B/GDF10) in lung cancer cells. Mol. Cancer.

[46] Pockwinse S.M., Kota K.P., Quaresma A.J., Imbalzano A.N., Lian J.B., van Wijnen A.J., Stein J.L., Stein G.S., Nickerson J.A. (2011). Live cell imaging of the cancer-related transcription factor RUNX2 during mitotic progression. J. Cell Physiol..

[47] Heinz S., Benner C., Spann N., Bertolino E., Lin Y.C., Laslo P., Cheng J.X., Murre C., Singh H., Glass C.K. (2010). Simple combinations of lineage-determining transcription factors prime cis-regulatory elements required for macrophage and B cell identities. Mol. Cell.

[48] Ghisletti S., Barozzi I., Mietton F., Polletti S., De Santa F., Venturini E., Gregory L., Lonie L., Chew A., Wei C.L. (2010). Identification and characterization of enhancers controlling the inflammatory gene expression program in macrophages. Immunity.

[49] Pio F., Kodandapani R., Ni C.Z., Shepard W., Klemsz M., McKercher S.R., Maki R.A., Ely K.R. (1996). New insights on DNA recognition by ets proteins from the crystal structure of the PU.1 ETS domain-DNA complex. J. Biol. Chem..

[50] Zhang P., Zhang X., Iwama A., Yu C., Smith K.A., Mueller B.U., Narravula S., Torbett B.E., Orkin S.H., Tenen D.G. (2000). PU.1 inhibits GATA-1 function and erythroid differentiation by blocking GATA-1 DNA binding. Blood.

[51] Stopka T., Amanatullah D.F., Papetti M., Skoultchi A.I. (2005). PU.1 inhibits the erythroid program by binding to GATA-1 on DNA and creating a repressive chromatin structure. EMBO J..

[52] Passner J.M., Ryoo H.D., Shen L., Mann R.S., Aggarwal A.K. (1999). Structure of a DNA-bound Ultrabithorax-Extradenticle homeodomain complex. Nature.

[53] Jabet C., Gitti R., Summers M.F., Wolberger C. (1999). NMR studies of the pbx1 TALE homeodomain protein free in solution and bound to DNA: Proposal for a mechanism of HoxB1-Pbx1-DNA complex assembly. J. Mol. Biol..

[54] Moens C.B., Selleri L. (2006). Hox cofactors in vertebrate development. Dev. Biol..

[55] Magnani L., Ballantyne E.B., Zhang X., Lupien M. (2011). PBX1 genomic pioneer function drives ERalpha signaling underlying progression in breast cancer. PLoS Genet..

[56] Lupien M., Eeckhoute J., Meyer C.A., Wang Q., Zhang Y., Li W., Carroll J.S., Liu X.S., Brown M. (2008). FoxA1 translates epigenetic signatures into enhancer-driven lineage-specific transcription. Cell.

[57] Burke L.J., Zhang R., Bartkuhn M., Tiwari V.K., Tavoosidana G., Kurukuti S., Weth C., Leers J., Galjart N., Ohlsson R., Renkawitz R. (2005). CTCF binding and higher order chromatin structure of the H19 locus are maintained in mitotic chromatin. EMBO J..

[58] Kennedy G., Sugden B. (2003). EBNA-1, a bifunctional transcriptional activator. Mol. Cell Biol..

[59] Sears J., Kolman J., Wahl G.M., Aiyar A. (2003). Metaphase chromosome tethering is necessary for the DNA synthesis and maintenance of oriP plasmids but is insufficient for transcription activation by Epstein-Barr nuclear antigen 1. J. Virol..

[60] Sears J., Ujihara M., Wong S., Ott C., Middeldorp J., Aiyar A. (2004). The amino terminus of Epstein-Barr Virus (EBV) nuclear antigen 1 contains AT hooks that facilitate the replication and partitioning of latent EBV genomes by tethering them to cellular chromosomes. J. Virol..

[61] Nayyar V.K., Shire K., Frappier L. (2009). Mitotic chromosome interactions of Epstein-Barr nuclear antigen 1 (EBNA1) and human EBNA1-binding protein 2 (EBP2). J. Cell Sci..

[62] Bochkarev A., Barwell J.A., Pfuetzner R.A., Bochkareva E., Frappier L., Edwards A.M. (1996). Crystal structure of the DNA-binding domain of the Epstein-Barr virus origin-binding protein, EBNA1, bound to DNA. Cell.

[63] Cruickshank J., Shire K., Davidson A.R., Edwards A.M., Frappier L. (2000). Two domains of the Epstein-Barr virus origin DNA-binding protein, EBNA1, orchestrate sequence-specific DNA binding. J. Biol. Chem..

[64] Marechal V., Dehee A., Chikhi-Brachet R., Piolot T., Coppey-Moisan M., Nicolas J.C. (1999). Mapping EBNA-1 domains involved in binding to metaphase chromosomes. J. Virol..

[65] Wu H., Ceccarelli D.F., Frappier L. (2000). The DNA segregation mechanism of Epstein-Barr virus nuclear antigen 1. EMBO Rep..

[66] Wu H., Kapoor P., Frappier L. (2002). Separation of the DNA replication, segregation, and transcriptional activation functions of Epstein-Barr nuclear antigen 1. J. Virol..

[67] Shire K., Ceccarelli D.F., Avolio-Hunter T.M., Frappier L. (1999). EBP2, a human protein that interacts with sequences of the Epstein-Barr virus nuclear antigen 1 important for plasmid maintenance. J. Virol..

[68] Kapoor P., Lavoie B.D., Frappier L. (2005). EBP2 plays a key role in Epstein-Barr virus mitotic segregation and is regulated by aurora family kinases. Mol. Cell Biol..

[69] Hung S.C., Kang M.S., Kieff E. (2001). Maintenance of Epstein-Barr virus (EBV) oriP-based episomes requires EBV-encoded nuclear antigen-1 chromosome-binding domains, which can be replaced by high-mobility group-I or histone H1. Proc. Natl. Acad. Sci. USA.

[70] Sugden B., Warren N. (1989). A promoter of Epstein-Barr virus that can function during latent infection can be transactivated by EBNA-1, a viral protein required for viral DNA replication during latent infection. J. Virol..

[71] Gahn T.A., Sugden B. (1995). An EBNA-1-dependent enhancer acts from a distance of 10 kilobase pairs to increase expression of the Epstein-Barr virus LMP gene. J. Virol..

[72] Ceccarelli D.F., Frappier L. (2000). Functional analyses of the EBNA1 origin DNA binding protein of Epstein-Barr virus. J. Virol..

[73] Mackey D., Middleton T., Sugden B. (1995). Multiple regions within EBNA1 can link DNAs. J. Virol..

[74] Mackey D., Sugden B. (1997). Studies on the mechanism of DNA linking by Epstein-Barr virus nuclear antigen 1. J. Biol. Chem..

[75] Frappier L., O’Donnell M. (1991). Epstein-Barr nuclear antigen 1 mediates a DNA loop within the latent replication origin of Epstein-Barr virus. Proc. Natl. Acad. Sci. USA.

[76] Goldsmith K., Bendell L., Frappier L. (1993). Identification of EBNA1 amino acid sequences required for the interaction of the functional elements of the Epstein-Barr virus latent origin of DNA replication. J. Virol..

[77] Mackey D., Sugden B. (1999). The linking regions of EBNA1 are essential for its support of replication and transcription. Mol. Cell Biol..

[78] Lu F., Wikramasinghe P., Norseen J., Tsai K., Wang P., Showe L., Davuluri R.V., Lieberman P.M. (2010). Genome-wide analysis of host-chromosome binding sites for Epstein-Barr Virus Nuclear Antigen 1 (EBNA1). Virol. J..

[79] Canaan A., Haviv I., Urban A.E., Schulz V.P., Hartman S., Zhang Z., Palejev D., Deisseroth A.B., Lacy J., Snyder M. (2009). EBNA1 regulates cellular gene expression by binding cellular promoters. Proc. Natl. Acad. Sci. USA.

[80] Dresang L.R., Vereide D.T., Sugden B. (2009). Identifying sites bound by Epstein-Barr virus nuclear antigen 1 (EBNA1) in the human genome: Defining a position-weighted matrix to predict sites bound by EBNA1 in viral genomes. J. Virol..

[81] d’Herouel A.F., Birgersdotter A., Werner M. (2010). FR-like EBNA1 binding repeats in the human genome. Virology.

[82] Altmann M., Pich D., Ruiss R., Wang J., Sugden B., Hammerschmidt W. (2006). Transcriptional activation by EBV nuclear antigen 1 is essential for the expression of EBV's transforming genes. Proc. Natl. Acad. Sci. USA.

[83] Aras S., Singh G., Johnston K., Foster T., Aiyar A. (2009). Zinc coordination is required for and regulates transcription activation by Epstein-Barr nuclear antigen 1. PLoS Pathog..

[84] Avolio-Hunter T.M., Lewis P.N., Frappier L. (2001). Epstein-Barr nuclear antigen 1 binds and destabilizes nucleosomes at the viral origin of latent DNA replication. Nucleic Acids Res..

[85] Avolio-Hunter T.M., Frappier L. (2003). EBNA1 efficiently assembles on chromatin containing the Epstein-Barr virus latent origin of replication. Virology.

[86] Zhou J., Chau C.M., Deng Z., Shiekhattar R., Spindler M.P., Schepers A., Lieberman P.M. (2005). Cell cycle regulation of chromatin at an origin of DNA replication. EMBO J..

[87] Chaudhuri B., Xu H., Todorov I., Dutta A., Yates J.L. (2001). Human DNA replication initiation factors, ORC and MCM, associate with oriP of Epstein-Barr virus. Proc. Natl. Acad. Sci. USA.

[88] Sarkari F., Sanchez-Alcaraz T., Wang S., Holowaty M.N., Sheng Y., Frappier L. (2009). EBNA1-mediated recruitment of a histone H2B deubiquitylating complex to the Epstein-Barr virus latent origin of DNA replication. PLoS Pathog..

[89] Hsieh C.L. (1999). Evidence that protein binding specifies sites of DNA demethylation. Mol. Cell Biol..

[90] Lin I.G., Tomzynski T.J., Ou Q., Hsieh C.L. (2000). Modulation of DNA binding protein affinity directly affects target site demethylation. Mol. Cell Biol..

[91] Ohno S., Luka J., Lindahl T., Klein G. (1977). Identification of a purified complement-fixing antigen as the Epstein-Barr-virus determined nuclear antigen (EBNA) by its binding to metaphase chromosomes. Proc. Natl. Acad. Sci. USA.

[92] Grogan E.A., Summers W.P., Dowling S., Shedd D., Gradoville L., Miller G. (1983). Two Epstein-Barr viral nuclear neoantigens distinguished by gene transfer, serology, and chromosome binding. Proc. Natl. Acad. Sci. USA.

[93] Kanda T., Kamiya M., Maruo S., Iwakiri D., Takada K. (2007). Symmetrical localization of extrachromosomally replicating viral genomes on sister chromatids. J. Cell Sci..

[94] Wilson J.B., Bell J.L., Levine A.J. (1996). Expression of Epstein-Barr virus nuclear antigen-1 induces B cell neoplasia in transgenic mice. EMBO J..

[95] Kang M.S., Lu H., Yasui T., Sharpe A., Warren H., Cahir-McFarland E., Bronson R., Hung S.C., Kieff E. (2005). Epstein-Barr virus nuclear antigen 1 does not induce lymphoma in transgenic FVB mice. Proc. Natl. Acad. Sci. USA.

[96] Kang M.S., Soni V., Bronson R., Kieff E. (2008). Epstein-Barr virus nuclear antigen 1 does not cause lymphoma in C57BL/6J mice. J. Virol..

